# Transcriptomic Challenges We Faced with Animal Models for Neurological Disorders

**DOI:** 10.3390/cimb48090857

**Published:** 2026-08-24

**Authors:** Dumitru A. Iacobas, Sanda Iacobas, Dennis Daniels

**Affiliations:** 1 Undergraduate Medical Academy, School of Public and Allied Health, Prairie View A&M University, Prairie View, TX 77446, USA; dedaniels@pvamu.edu; 2 D.P. Purpura Department of Neuroscience, Albert Einstein College of Medicine, Bronx, NY 10461, USA; 3 Genomics Core, Department of Pathology, New York Medical College, Valhalla, NY 10565, USA; sandaiacobas@gmail.com; 4 Department Humanities in Medicine, College of Medicine, Texas A&M University System Health Science Center, College Station, TX 77845, USA

**Keywords:** AT-EAE, cerebral malaria, cerebral palsy, CMT1X, epilepsy, glaucoma, infantile spasms, intraventricular hemorrhage, neuropsychiatric lupus erythematosus, ODDD

## Abstract

Simulation of a human neurological disease on an animal model has the advantage of allowing control and manipulation of most of the regulating factors and producing real biological replicas while, beyond several common traits, every human is dynamic and unique. Moreover, one can explore novel therapeutic strategies on animals before asking permission to apply them to humans. Nevertheless, experimental outcomes depend on species, strain, sex, age, hormonal status, diet, exposure to hypoxia, toxins, radiation, external stimuli, stress, and housing conditions. Further complications stem from tissue hetero-cellularity, technological constraints, computational complexity and difficulties integrating the experimental results into a coherent biological picture. Moreover, most diseases are multi-factorial and associated with altered structure and/or expression of several genes. A major problem with genetically engineered animals is that together with the targeted gene(s), numerous other genes are mutated and/or regulated, owing to their interlinkage in functional pathways. This experience-based methodological commentary presents the challenges, relevance and limitations of the mouse, rat and rabbit models we used to decipher the transcriptomic alterations associated with several neurological disorders. Links to publicly accessible databases presenting experimental protocols and expression profiles are provided for readers interested in reanalyzing our data and comparing them with others’ results.

## 1. Introduction

In addition to common disease indicators related to memory, cognition, behavior, and senses, medical statistics categorizes neurological patients into large groups according to race [1], sex [2], age group [3], geography [4], and climate [5]. However, there are numerous other non-negligible influential factors, like smoking [6], alcohol [7] and drug [8] consumption habits, medical history [9,10], diet [11], exposure to stress [12] and toxins [13], whose dynamic combination is unique for each human. Even monozygotic twins might be differently affected by the same disease, each of them experiencing distinct severity and development course and treatment outcomes [14,15].

Beyond locally imposed moral and religious rules, medical research on human subjects must also respect the ethical principles included in the Declaration of Helsinki [16] and the additional constraints imposed by the local Institutional Review Board (IRB). These mandatory rules limit exploration of molecular phenomena to tissues removed in routine surgery (eventually spread into immortalized cell cultures), blood and body waste, imaging and non-invasive investigations. Postmortem human tissue samples purchased from accredited biobanks are free of most ethical constraints but still represent a narrow spectrum of pathologies [17,18].

Therefore, one needs to replicate human diseases into animal models that, although restricted to protocols approved by the local Institutional Animal Care and Use Committee (IACUC), still allow studies that would never be acceptable to conduct on living humans. IACUC should adhere to internationally recognized ethical principles [19] and align with the Guide for the Care and Use of Laboratory Animals (e.g., [20]). Our expertise on neurological diseases is with animal models, although in cancer research, we have also profiled surgically removed thyroid, prostate, lung and kidney tumors from living human patients. Although there is no way to exactly reproduce a human disease on an animal model, one may assume that certain recipes for physiological phenomena and their alterations are shared with some other mammals and remain unchanged during evolution. Moreover, a non-negligible advantage of the animal model is that the investigator has full control of the experimental conditions, while each human is affected by a combination of favoring factors that are never repeatable and are mostly out of our control.

Here, we present an experience-based methodological commentary (EBMC) that details the challenges our labs faced in developing and investigating animal models for some neurological disorders over many years of research. On reviewing the animal models, we had to discuss the limits and technical noise of the experimental approaches to both generate and investigate them, as well as of the ways to analyze the data. However, most of the issues presented here are common to all genomic studies on animal models.

The challenges are illustrated through our genomic investigations on hippocampus regions, hypothalamic arcuate and periventricular nuclei, frontal cortex, spinal cord, striatum, dorsal root ganglia, retina, and primary, immortalized and genetically engineered neurons, astrocytes, and oligodendrocytes. The cells and tissues were collected from mouse, rat and rabbit colonies, maintained in-house, that modeled astrocytoma, epilepsies, glaucoma, cerebral malaria, multiple sclerosis, neuroblastoma, neuropsychiatric lupus erythematosus, occulo-dento-digital dysplasia, and X-linked Charcot–Marie–Tooth diseases. We were also involved in confocal imaging of calcium signaling in mouse astrocytoma, behavioral studies of epileptic rats, and electrophysiological experiments on various components of the nervous systems of mice, rats, rabbits, earthworms, and frogs. In addition to caring for animal colonies and cell cultures, improving technology, and performing molecular experiments with optimized wet protocols, we developed mathematically advanced algorithms and software, and acquired bioinformatic expertise.

Beyond citing our own publications and transcriptomic databases, supporting literature in this EBMC was selected according to the following criteria: (i) first and/or most relevant animal model(s) of the discussed disorder, (ii) first and/or most advanced experimental method(s) to generate the model, (iii) first and/or most accurate method(s) to investigate the model, (iv) first and/or most advanced method(s) to analyze the experimental data. Controversial animal models, experimental methods and analytical procedures were excluded from the cited literature. Nevertheless, given the wide palette of the discussed models, it is practically impossible to cite all of the relevant literature, and potential major citations are inherently missing.

## 2. Major Determinants of Animal Models

All molecular characteristics of an animal model depend on species [21,22], strain [23,24,25], sex [26,27,28,29,30], age/developmental stage [31,32,33], tissue [34,35], and explored region [36] and the way the model was generated (by genetic engineering (e.g., [37,38]), or by chemical (e.g., [39]), hormonal (e.g., [40]), parasitical (e.g., [41]), or mechanical (e.g., [27,28]) induction). They are modulated by diet [42,43,44,45,46], exposure to oxygen deprivation [47,48], toxins [49,50,51], radiation [52], and external stimuli [53,54,55,56]. Not negligible are the hormonal status [57,58,59,60,61,62,63], disease history [64,65] and treatment [66,67], and even exposure to microgravity [68,69]. We proved that neuronal status is also strongly dependent on the pattern of action potential [55,70]. Moreover, the subcellular localization of certain proteins not only differs between sexes but also changes during the estrogen cycle [61,71,72,73], making female animal models much more difficult to manage and interpret than their male counterparts.

**Figure 1:**
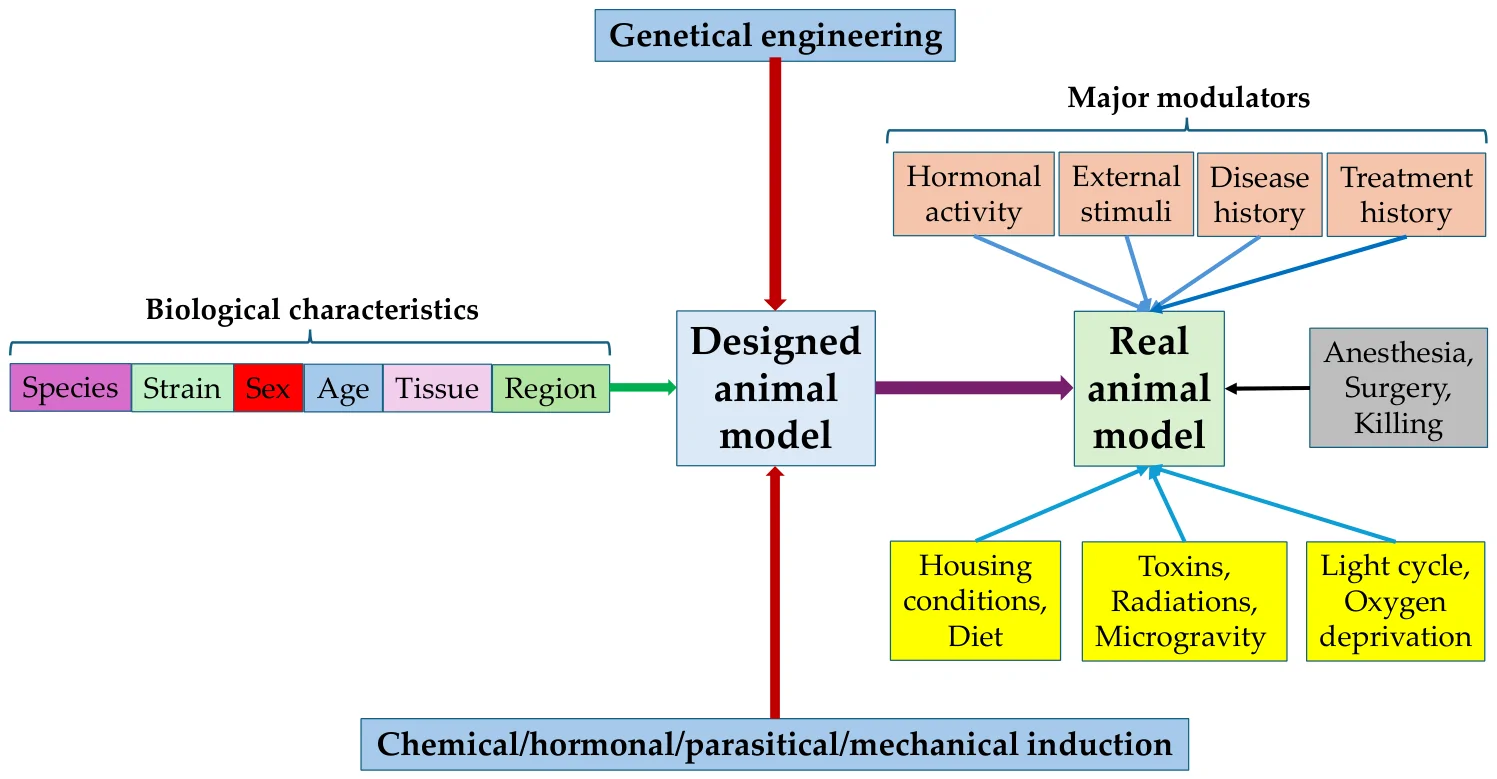
Characteristics and part of the major modulators of an animal model. Arrows go from input to output. Background colors added to distinguish among input and types.

Therefore, choosing and handling the right animal model [74] is far from an easy task. Furthermore, sanitary precautions and housing conditions (e.g., distribution of cages and vicinity of other caged animals [75,76], chow and water abundance and quality, environmental temperature, humidity, and day–night lighting cycle [77]) are also major modulators of the experimental results. Not negligible are the neurological effects of the anesthesia used for surgery (usually ketamine/xylazine 70/7 mg/kg)) and painless sacrifice of the animal. For instance, the intraperitoneal injection of urethane we used to study the neuro-transcriptomic effects of microgravity [68] desynchronizes the electric waves in the hippocampal dentate gyrus (DG) [78]. Therefore, to minimize the anesthesia effects on the nervous system, in other experiments we used either carbon dioxide [79,80] or just decapitation. Figure 1 schematizes the major determinants of a real animal model.

However, even if all animal characteristics were hypothetically identical, there is still biological variability due to the stochasticity of biological responses. The variability also extends to samples collected from different regions of the same tissue, as we found by profiling pathologically equally graded cancer nodules in surgically removed prostate [81,82] and kidney [83,84] cancer tumors from the same individuals. This transcriptomic heterogeneity results from the coexistence of several stem cell clones with similar yet nonidentical phenotypes within the same region, potentially generated by exposure to different local conditions [85,86].

Increasing evidence shows that the molecular characteristics of individual cells depend on cellular environment in a heterogeneous tissue [87,88] and change dramatically when studied outside of their original tissue. We demonstrated the impact of cellular environment on major cellular functional pathways by profiling the transcriptomes of astrocytes and oligodendrocytes when co-cultured in insert systems vs. cultured separately [89,90,91,92,93]. For instance, we found that proximity of even non-touching oligodendrocyte regulates numerous astrocyte genes involved in controlling the neuronal synaptic transmission and profoundly remodels most of the signaling pathways, actin-cytoskeleton, autophagy, cell cycle, and circadian rhythm. On the oligodendrocyte site, the proximity of astrocytes activates the gene machinery of myelination and other functional pathways.

The characteristics of the specimen studied are also strongly influenced by the culture preparation technique [94,95,96] and applied stimuli [54,97,98,99], hormones [100,101,102] and chemical treatment [103,104]. In some investigations [89,90,91,92,93], we have used immortalized mouse oligodendroglial precursor cell lines (Oli-neu) obtained through the standard protocol [105,106], while in others we used primary cultures of mouse and rat neurons, astrocytes, oligodendrocytes and microglia [54,94]. Therefore, we can affirm that, although preferred for their stability, immortalized cell lines [107,108,109] have substantially modified physiology with respect to the primary cells. The purity of cell culture is extremely important, not only because the result would be averaged expression levels over all present phenotypes, but the “infectant” might interact and thus modify the transcriptome of the investigated cells. However, even in pure cultures, cells do not behave identically. For instance, in cultured rat β-pancreatic cells, we found that only part of them expressed connexin 36, and in cultured HL-1 cardiomyocytes, some cells were contracting spasmodically, while others were not (Iacobas, unpublished observations). When possible, it is also preferable to synchronize the cultured cells and profile at various stages of the cell cycle to detect the dynamics of the transcriptomic topology. As such, one should be careful when extending the findings from either primary or immortalized cell cultures to the original hetero-cellular tissue.

## 3. Genetically Engineered Animal Models

### 3.1. General Considerations

It is a widespread belief that many diseases are frequently associated with altered sequence (e.g., [110,111,112]) and/or expression level (e.g., [113,114,115,116]) of certain critical genes, termed (gene) biomarker(s) (e.g., [117,118,119,120]). Therefore, it is assumed that, by engineering the same alteration in the genome of an animal, one might reproduce the key features of that human disease (e.g., [121,122]). It is also assumed and already included in clinical trials that a cure may be provided by restoring the normal sequence/expression level of the biomarker gene(s) (e.g., [123,124]).

The problem is that many disorders are multifactorial, involving alteration of two or more genes. For instance, mutations of six genes (*SNCA*, *LRRK2*, *Parkin*, *PINK1*, *DJ-1*, *ATP13A2*) are blamed for Parkinson disease [125], while NF-κB, NLRP3 inflammasome, and mTOR signaling pathways are significantly altered following spinal cord injury [126]. A few selected multi-gene diseases were simulated in double knockout mouse models, such as the *Gfap*^−/−^*Vim*^−/−^ mouse with attenuated glial scar formation post-stroke [127]. Moreover, effective mutations can be located within several exons, such as the 65 known mutations of *GJA1* identified as responsible for autosomal-dominant ODDD [128].

Despite being adopted by most genomicists because of statistical significance in large cohort studies, the biomarker paradigm has major flaws [129]. When even pathologically graded cancer nodules from the same tumor exhibit substantial differences in transcriptomic topologies [81,83], it is hard to accept that distinct individuals exhibiting similar symptoms should have the same set of gene mutations and/or transcriptomic signature. However, biomarkers remain useful in diagnosis, stratification, prognosis, and mechanistic studies when interpreted appropriately. Hence, we have proposed replacing the gene biomarker paradigm with the genomic fabric paradigm (GFP) [47] that provides the most theoretically possible comprehensive characterization of the transcriptome. Moreover, GFP allows identification of personalized gene master regulators [130] whose silencing might selectively kill the cells they command in a hetero-cellular tissue.

The main challenge with the use of genetically engineered animals and cells is that the targeted mutation goes over the ~3 million other mutations present in every cell at any given time. About one in every thousand nucleotides is randomly mutated just because of the stochastic nature of the chemical reactions involved in DNA replication [131], although most of these mutations have practically no effect because they occur in non-coding regions. Moreover, in addition to the gene whose expression level is experimentally altered, expressions of hundreds of other genes are also regulated through their interlinkages in functional pathways. To make things much more difficult, the combination of the affected genes is not only unique to everyone and even to each histopathologically distinct region of the tissue but also changes over time. Furthermore, each region exhibits distinct strengths in the homeostatic control of transcript abundances, gene networks, and functional pathways. We encountered these difficulties in all genetically engineered animal models (described below) used in our neuro-genomics studies.

However, beyond trying to reproduce the main feature of the human disease, most genetically engineered animal models aim to elucidate certain molecular mechanisms associated with an experimentally altered sequence(s) and/or expression level(s) of one or a few genes.

### 3.2. Occulo-Dento-Digital Dysplasia (ODDD)

ODDD, first described by Lohman in 1920 [132], is believed to be caused by mutations in the *GJA1* (gap junction alpha 1) gene, located at 6q22.31, that encodes connexin 43 (Cx43), the most important gap junction channel forming protein in astrocytes [128,133,134]. Neurological manifestations of ODDD include isolated ataxia combined with spasticity [135,136], microphthalmia, microcornea, glaucoma, cataracts, and optic neuropathy [137]. ODDD was simulated in genetically engineered C57Bl/6j, C3H/HeJ and FVB mice (e.g., [138]). I130T/+ and G60S/+ mutations of *Gja1* reproduce most of the ODDD features [139,140,141] in mouse models.

There are 41 available clinical tests and 74 molecular genetics tests for *GJA1* mutations responsible for ODDD [142] and several publicly accessible gene expression databases on various regions of the nervous system of *Gja1* KO mice (e.g., [143,144,145,146]). We profiled the gene expression in brains and cortical astrocytes from C57Bl/6j and 129/SvEv mice whose *Gja1* gene was: (i) knocked out (Cx43KO, *Gja1^−^*^/*−*^) through homologous recombination [147,148,149,150], (ii) conditionally knocked out with hGFAP-Cre:Cx43f/f (method in [31]) only in the brain [23], or (iii) knocked down through siRNA [94]. In addition to Cx43*^−^*^/*−*^ mice, we also profiled brains and cortical astrocytes from Cx43^+/*−*^ mice [151,152] and performed several other transcriptomic experiments with truncated carboxy terminal of Cx43 (CT-Cx43) on various loci [153]. However, although the brain looks anatomically normal, the Cx43KO mouse is not a good model for ODDD since the mutant dies at birth because of a developmental heart abnormality [154]. Therefore, we had to profile brains of *Gja1*^−/−^ either just after birth or even taken out from their mothers with C-section on day G17. Although the preterm and just-born mice with a genetically altered *Gja1* did not exhibit ODDD features, the experiments provided valuable information about the major roles played by *Gja1* in modulating several functional pathways and its interaction with membrane purinergic receptors [155]. Moreover, we found that the transcriptomic alterations strongly depended on the mouse strain (e.g., C57Bl/6 vs. 129/SvEv) [23].

### 3.3. X-Linked Charcot–Marie–Tooth Syndrome Type (CMT1X)

CMT1X is a demyelinating disorder characterized by muscle weakness and sensory neuropathy [156,157]. It is believed that CMT1X is caused by loss-of-function mutations affecting the *GJB1* (gap junction beta 1) gene, located on chromosome X, that encodes connexin 32 (Cx32) the gap junction channel forming protein in oligodendrocytes (brain) and Schwann cells (peripheral nervous system) [158]. Over 400 mutations of *GJB1* were already reported in CMT1X patients [158], stimulating gene therapy research (e.g., [159]. Several CMT1X-causing missense mutations of *GJB1* have been identified so far, including p.Arg15Trp, p.Val63Ile, p.Leu89Val, p.Ala96Gly, p.Arg107Trp, p.Arg142Gln, p.Arg164Trp, p.Arg164Gln, p.Pro172Ala, p.Asn205Ser, p.Val13Glu, p.Glu186Gly, and p.Met194Ile [160]. The disease was reproduced in several mouse models (e.g., [161]) by knocking out *Gjb1* [162] or by CRISPR/Cas9 genome editing to induce mutations in p.T55I, p.R75W [163], or R15Q [164] in *Gjb1*. Although over 100 other genes were blamed for CMT1X [165], *GJB1* remains the main suspect and animal modeling is focusing on manipulating this gene.

Because males have only one X chromosome, while females have two, *GJB1* pathological alterations occur more frequently, at younger age and with higher severity in men than in women with Cx32 deficiency [166]. We compared the brain transcriptomes of *Gjb1^−/^*and wild-type C57Bl/6j male mice and found that together with *Gjb1*, hundreds of other genes were significantly regulated [150]. Moreover, the significant overlap between the brain regulomes of *Gja1*^−/−^ and *Gjb1*^−/−^ mice indicates a pan-glial transcriptomic continuity in the brain [167,168], although the two genes are expressed in different brain cells and there is no known overlap between the ODDD and CMT1X syndromes.

### 3.4. Temporal Lobe Epilepsy (TLE)

TLE is a group of chronic brain disorders characterized by recurring seizures (abnormal, paroxysmal changes in the electrical activity) of the brain [169], excitotoxicity, neuronal loss and cognitive decline [170,171]. Frequently used rat models are obtained by administration of a chemoconvulsant like pilocarpine [172], pentylenetetrazol [173], kainic acid [174] or flurothyl [175]. Other models are obtained by traumatic brain injury [171], electrical kindling [173] and genetic manipulation of the sodium channel gene *SCNA1* [175]. The efficiency of cannabidiol treatment of TLE was tested on a mouse kindling model [176]. There are several available gene expression databases obtained by profiling selected brain regions from TLE animal models that can be used for further analyses (e.g., ref. [177] for a kainic acid model, refs. [178,179] for a lithium-pilocarpine model, refs. [180,181] for intrahippocampal electrical kindling and systemic kainic acid models).

It was reported that *GJD2* (gap junction delta2), which encodes connexin 36 (Cx36), the main neuronal gap junction channel forming protein in neurons (microglia and pancreatic cells), plays a major role in epilepsy [182,183]. This finding stimulated the use of quinine, a blocker of the interneuronal Cx36 gap junction channel, as an effective suppressor of seizures [184,185]. Our transcriptomic study of *Gjd2*^−/−^ (formerly known as *Gja9*^−/−^) and *Gjd2*^+/−^ mouse brains revealed hundreds of other genes being significantly regulated in addition to *Gjd2.* We also found that the *Gjd2*^−/−^ mouse brain regulome was largely different from those of the *Gja^−/−^* and *Gjb^−/−^* mice [147,150], indicating a lack of transcriptomic interaction between neurons and glial cells.

### 3.5. Juvenile Myoclonic Epilepsy (JME)

Genome-wide association studies identified the loci 4p12, 8q23.1 and 16p11.2 [186] as the most susceptible for JME, a subsyndrome of idiopathic generalized epilepsy [187] that starts in adolescence [188]. JME was simulated in several transgenic mouse models including Efhc1 (EF-hand domain containing 1)-deficient [189], Kcnc1-p.Arg320His/+ [190], *Brd2*^+/−^ [191] and *Cstb*^−/−^ [192,193]. Recent RNA sequencing data proved that *EFHC1* regulates neurogenesis [194]. Interestingly, none of the mutations of *CILK1* (ciliogenesis associated kinase 1)/ICK (intestinal cell kinase), blamed for JME in humans [195,196], reproduced the JME phenotype in mice [195], questioning the validity of this gene biomarker. The diversity of the animal models is justified by the multi-gene JME etiology. In recent years, bromodomain containing 2 (*Brd2*), a putative nuclear transcription regulator, is considered a major JME susceptibility gene [197,198,199]. The haploinsufficiency in a bromodomain containing 2 (*Brd2*^+/−^) JME mouse model revealed GABAneuron deficit [200] and sex-specific behavioral traits [201] that we explain by the observed sex differences in the networking of neurotransmission genes in our transcriptomic study on a *Brd2*^+/−^ mouse model [202,203,204].

### 3.6. Neuropsychiatric Lupus Erythematosus (NPSLE)

Patients with NPSLE present cognitive impairment, psychosis, anxiety, mood, and movement disorders, confused state, memory loss, seizures, stroke, and headache [205]. The interaction of the tumor necrosis factor (TNF)-like weak inducer of apoptosis (*TWEAK* or *TNFSF12*) with its cognate receptor, *FN14* (*TNFRSF12A*, expressed in astrocytes, microglia, brain microvascular endothelial cells, and neurons), is believed to activate pro-inflammatory cytokine production [206]. Several murine models have been develo4) Reference was always the corresponding wildtype to investigate the molecular mechanisms of NPSLE (cited in [207]), including NZBNZW (Crossed New Zeeland Black and white mice) and the popular MRL/lpr [208,209,210]. Currently in use are also the tetramethylpentadecane (known also as pristane)-induced [211] and dLckCre [212] NPSLE mouse models.

We profiled the transcriptomes of brain cortices and hippocampi of MRL^/+^, MRL/lpr and MRL/lpr-Fn14 knockout (Fn14ko backcross generation #8) adult female mice, all sacrificed at diestrus of the estrogen cycle to minimize the biological variability caused by hormonal activity. The experiments revealed significant alterations in the chemokine and PI3K/AKT signaling pathways [213,214] and an interesting link between neuroinflammation and neurodegeneration [215,216].

Table 1 summarizes the main features (species, train, sex, age, tissue), limitations and relevancies of our genetically engineered animal models of neurological disorders. Notes: (1) All experiments respected MIAME standards [217]; (2) Any experimental manipulation of a gene triggered mutations and expression regulation of numerous other genes; (3) Each condition/tissue was profiled in 4 biological replicates collected from animals of the same litter; (4) Reference was always the corresponding wildtype; (5) When needed, several brain regions/cells were collected from the same animal.

## 4. Induced Animal Models of Neurological Diseases

### 4.1. General Considerations

Numerous chemical, hormonal, parasitical and mechanical (to name just a few) methods to induce a neurological disease in an animal model have been developed so far. Nevertheless, despite the symptoms’ similarities with human disorders, the used inductor is rarely the cause of that affliction in a real patient. However, the induced models provide valuable tools to investigate possible altered mechanisms responsible for the observed symptoms and explore therapeutic avenues.

### 4.2. Multiple Sclerosis (MS)

MS is an inflammatory demyelinating disease that alters the neuronal circuits in the brain and spinal cord, resulting in paralysis and death [218,219]. Current MS therapy includes administration of immunomodulators and immunosuppressants but it just alleviates the symptoms and reduces relapse frequency without stopping progression of disability [220]. MS symptoms were satisfactorily replicated in rodents’ adoptive transfer experimental autoimmune encephalomyelitis (AT-EAE) [221,222]. AT-EAE is induced by injecting myelin basic protein (Mbp) dissolved in sterile phosphate-buffered saline and emulsified with incomplete Freund’s adjuvant supplemented with *Mycobacterium tuberculosis* emulsion in the rodent spinal cord [223]. The disease develops after 8–10 days, with the animals manifesting ascending paralysis, starting with the hind limbs. There are several publicly accessible gene expression databases on this model that can be used for further transcriptomic analyses [224,225,226,227].

Our study on spinal cords of SJL/J adult, clinical index 4 (hind- and front-limb paralysis) female AT-EAE mice revealed that *Gja1* is downregulated, and its downregulation correlates with a substantial increase in the populations of dystrophic neurons and monocytes [228]. We also found that, together with the immune response, the expression control and the coordination of several genes included in the functional pathways Ca^2+^-signaling, cell cycle, cytoskeleton, energy-metabolism, RNA-processing, and transport of ions and small molecules were also altered [229,230].

### 4.3. Glaucoma (GL)

GL is a group of eye diseases that lead to blindness that is caused by degeneration of the retinal ganglion cells (RGCs), sometimes triggered by damage to the optic nerve [231]. The disease was induced in domestic chicks by exposure to continuous intense light [232], in rabbits by lensectomy, vitrectomy, and transvitreal photocoagulation of the ciliary processes [233] and in rats by injection of magnetic microspheres [234] or hydrogel [235] into the anterior chamber. It was also obtained in rats by external ocular compression through circumlimbal suture [236] or by crushing the optic nerve [237], and in mice by subretinal or intravitreal injections of fluorescently labeled mitochondria [238]. Gene expression data from the retinas and corneas of glaucomatous animal models, including a transcriptomic atlas [239], can be found in the Gene Expression Omnibus (GEO) of the National Center for Biotechnology Information (e.g., [240,241,242,243]).

We explored the retinal transcriptomes of adult Lister Hooded rats two weeks after optic nerve crush (ONC) and compared them with those of sham-operated controls to test similar surgical stress. Experiments have shown about a 60% reduction in the number of viable RGCs and significant alterations in the expression level, control and networking of genes involved in the complement cascade and Notch signaling functional pathways [244,245]. However, excepting exposure to intense light, the other methods to induce glaucoma in animal models are rarely the cause of the disease in humans and therefore RGC degeneration might follow a different trajectory [246]. Moreover, in addition to the species, the relevance of the animal model also depends on the rodent strain [247].

### 4.4. Catamenial Epilepsy (CE)

CE, also known as menstrual seizures, is a cyclical change of seizure frequency at various stages of the menstrual cycle [248] owing to the interdependence between β-estradiol concentration and synaptic transmission. CE was partially induced in rodent female models by extended exposure to high levels of progesterone and estrogens followed by rapid decline [249] or by administration of exogenous hormones in ovariectomized rats [250]. Recent models use optogenetics consisting in stimulation of membrane ion channels of parvalbumin-positive interneurons by blue light pulses [251].

To quantify the influence of the sex hormone on the epilepsy development, we profiled the DG transcriptomes of intact and ovariectomized (OVX) 8–9-week-old female Sprague-Dawley rats with kainic acid-induced status epilepticus (OVX-KA model). Kainic acid was injected through mini-osmotic pump implants [252] and the success of castration was confirmed by measurement of vaginal impedance. The estrogen protection of neurotransmission was quantified by comparing the DG transcriptomes of OVX females that received 17β-estradiol benzoate and the OVX females that received only sterile peanut oil with the DG transcriptome of the intact females [57]. From the perspective of estrogen production, OVX female rats recapitulate the menopausal state despite being forced into a young female state, whereas in women, it occurs naturally at mature ages. However, the epilepsy in menopausal women is not triggered by kainic acid, so the dynamic of the disease progression in humans is most likely different from what was observed in rats [253].

### 4.5. Infantile Spasms (IS)

IS (epileptic spasms during infancy or West syndrome [254]) starts in the first year of life, with the infant presenting stereotypical spasms, chaotic brain hypsarrhythmia and developmental delay [255]. Most likely caused by hypothalamic dysfunction, IS infants have decreased concentrations of adrenocorticotrophic hormone (ACTH) and cortisol in cerebrospinal fluid. Therefore, ACTH is the FDA-approved first-line treatment of IS [256]. There are several genetically engineered mouse models of IS in use, including: Arx (Aristaless-related homeobox) Knock-In (Arx(GCG)10+7), Arx Conditional Knock-Out (cKO), APC (Adenomatous polyposis coli) cKO, and Tsc1+/(Tuberous sclerosis 1) [257,258,259,260]. IS was also induced in developing rats by infusing tetrodotoxin (TTX) into the hippocampus [261].

We preferred the “Two-hit IS” model where the disorder is triggered by postnatally administering N-methyl-D-aspartic acid (NMDA) to rat pups prenatally primed with betamethasone. The model is based on the observation that prenatal exposure to corticosteroids like betamethasone in difficult pregnancy affects children’s mental development [262,263]. We explored the transcriptomic effects of prenatal (G15) intraperitoneal injection with betamethasone on synaptic transmission in the prefrontal cortex and in the arcuate and paraventricular hypothalamic nuclei of Sprague-Dawley rats of both sexes [264,265]. From postnatal day 12 (P12) to P15, male and female pups who prenatally received either betamethasone or saline were injected with either NMDA to trigger the spasms or just saline to experience similar injection stress [266]. The IS-positive rats were then randomly divided into 3 treatment groups: saline, ACTH or PMX53 (a potent, selective, cyclic hexapeptide, C5ar1 antagonist [267]). We found a significant sex dichotomy with males exhibiting more transcriptomic alterations of the (glutamatergic, GABAergic, cholinergic, dopaminergic, and serotonergic) synaptic transmission but also more efficient recovery following the anti-inflammatory treatment [66,264,265,268,269]. Importantly, the transcriptomic effects were significantly different in paraventricular than in the acuate nuclei collected from the same animals, indicating different regional brain sensitivity in developing this neurodegenerative disease [29]. Our studies confirmed the therapeutical value of PMX53 for neurodegenerative diseases [270], although NMDA administration is not the real cause of human IS.

### 4.6. Germinal Matrix Hemorrhage—Intraventricular Hemorrhage (IVH)

IVH, a major neurologic complication of 20% premature (<1500 g at birth) infants [271], is responsible for brain injury (including post-hemorrhagic hydrocephalus), cerebral palsy (10%), and mental retardation [272]. IVH has been induced so far in mouse [273,274,275,276], rat [277,278,279,280] and rabbit [281,282,283] animal models.

We used the standard protocol for rabbits delivered prematurely by C-section on G29 (32 days full-term) that were injected intraperitoneally 3 h postnatally with either 50% glycerol (6.5 g/kg) to trigger the IVH or saline for control reference [284]. Presence and severity of IVH were assessed 24 h postinjection by head ultrasound. Oligodendrocyte precursor cells were isolated from coronal slices cut at the level of the head of the caudate nucleus from the frontoparietal region. The “IVH-glycerol” rabbit model was used to determine the efficacy of the peroxisome proliferator activated receptor-γ (PPAR-γ) in enhancing myelination, and the efficacy of the 17β-estradiol treatment to restore the hippocampal dentate gyrus development [285,286]. We found that estrogen treatment increases the number of calbindin interneurons and prox1 neurons in the hippocampus dentate gyrus [287].

### 4.7. Cerebral Palsy (CP)

Children with cerebral palsy (CP) [288] present ataxia (lack of muscle coordination in voluntary movement), spasticity (stiff or tight muscles and exaggerated reflexes), weakness in one or more arms or legs, walking on the toes, a crouched gait, or a “scissored” gait [289]. Postnatal administration of glucocorticoids (GCs) in premature infants for the treatment of lung disease was associated with CP and neurodevelopmental delay [290]. CP was induced in mouse [291,292], rat [293,294] and rabbit [295,296].

We studied the effects of postnatal administration of glucocorticoids dexamethasone and betamethasone on the preterm (G29) rabbit forebrain (“CP-rabbit” model). The experiments have shown that postnatal GC induces hypomyelination, gliosis and neurologic deficits [297,298].

### 4.8. Cerebral Malaria (CM)

CM, a severe neurological manifestation of *Plasmodium falciparum* infection, is associated in 50% of cases with cognition deficit, memory impairment, visual ataxia, seizures, hemiplegia, psychiatric disorders, and deficient motor coordination [299,300,301]. The most popular mouse model is obtained through intraperitoneal injection of blood infected with *Plasmodium berghei* ANKA, a single-celled parasite from the subgenus Vinckeia [302,303,304,305], whose similarities and differences with the simulated human disorder were analyzed in a PloS Pathology published study [306].

Our gene expression study with CM simulated in 5-week-old C57BL/6j female mice [79,80] infected with *Plasmodium berghei* ANKA revealed substantial transcriptomic alteration of genes involved in chromatin remodeling, cell development, negative regulation of apoptosis, lipid metabolism, hydrolase activity, walking, and regulation of muscle contraction. The main limitation of our study was profiling the entire brain, without considering brain regional differences.

Table 2 summarizes the main features (species, train, sex, age, tissue), limitations and relevancies of our animal models with inducted neurological disorders. Notes: (1) All experiments respected MIAME standards [218]; (2) Each condition/tissue was profiled in 4 biological replicates collected from animals of the same litter; (3) Reference was always the corresponding healthy counterpart; (4) When needed, several brain regions/cells were collected from the same animal.

## 5. Experimental Issues

### 5.1. Technical Noise

From sample collection to result delivery, any molecular investigation is affected by technology’s limited resolution in detecting small concentrations, distorted outcomes due to saturation at high concentrations, and inherent technical noise that produces nonuniform background signals. Both resolution and saturation limits are continually improved by technological advancements. They can also be optimized through sample preparation protocols (adjusting sample dilution and the amounts of fluorescent markers), more efficient processing kits, and recalibration of scanning parameters. Although not explicitly discussed in most publications and rarely estimated by costly profiling of technical replicates, technical noise remains the main source of errors of transcriptomic studies.

Investigators now have the ability to choose from a wide diversity of transcriptomic platforms, of which large groups of next-generation RNA sequencing, microarrays and qRT-PCR are the most popular.

RNA sequencing is based on converting the mRNAs obtained by rRNA depletion/mRNA enrichment of total RNA extracted from the tissue of interest into fragments of complementary DNAs (cDNAs), which are then aligned to standard or de novo genome. There are several potential noise-generating steps in library preparation, clustering and sequencing. Part of it is caused by the impossibility of fully eliminating hybridization errors and washing out the fragments that are no longer needed.

Over one hundred single-cell sequencing methods have been developed so far to determine separately the omics of the several cell types composing a tissue (e.g., [307,308,309,310]) but it is difficult to estimate how much the separation method of cell phenotypes alters the real transcriptome. Moreover, transcript count appears sparse in some platforms, which might bias the expression ratios between two cell phenotypes (Iacobas, unpublished observations). Two- and three-dimensional coculture systems [311,312,313,314], where two or more cell types are grown with or without direct physical contact, are now used by many groups trying to understand the intricacy of nervous system physiopathology [315,316,317]. Nonetheless, integrating multicellular features of a hetero-cellular tissue into a mathematical model faces enormous theoretical difficulties and requires powerful computer techniques, like those used in machine learning approaches [317].

Microarray technology relies on selective hybridization of fluorescently labeled cDNAs obtained by reverse transcribing of total RNA with tens of thousands of ~1000 bp DNA clones (the cDNA method) or with 50–70 mer oligonucleotide sequences spotted on the treated surface of a glass slide. Because of this, the microarray can detect only the transcripts that stably hybridize to the spotted sequences and are not removed by the washing. Moreover, inherent slight differences on the deposited cDNAs or oligonucleotide amounts occur among the microarray domains printed on the glass slide with different pins that would bias the results if not corrected by an appropriate normalization procedure, for which we developed an original algorithm.

The protocol for quantitative reverse transcription polymerase chain reaction (qRT-PCR) involves reverse transcription of RNA into cDNA, followed by quantitative PCR to measure RNA levels by comparing to a standard curve. As such, qRT-PCR is affected by technical noise during RNA extraction, purification, cDNA conversion, and amplification.

A gene may be transcribed into numerous mRNAs differing from each other by what exons were used, how much their normal succession was preserved, whether their nucleotide sequences were integral or truncated, and whether portions of exons from other genes were translocated. By evidence, local conditions are neither uniform nor constant, depending on various physical and chemical interactions at the site of transcription. Therefore, the distribution of gene transcripts depends on the animal strain, sex, tissue, disease development, and response to treatment and/or other external stimuli. As a result, a microarray study might miss some very important transcripts in the investigated tissue and condition. On the other side, increasing the number of distinct transcripts picked by the RNA sequencing is limited by the increasing time and cost of the sequencing.

**Figure 2:**
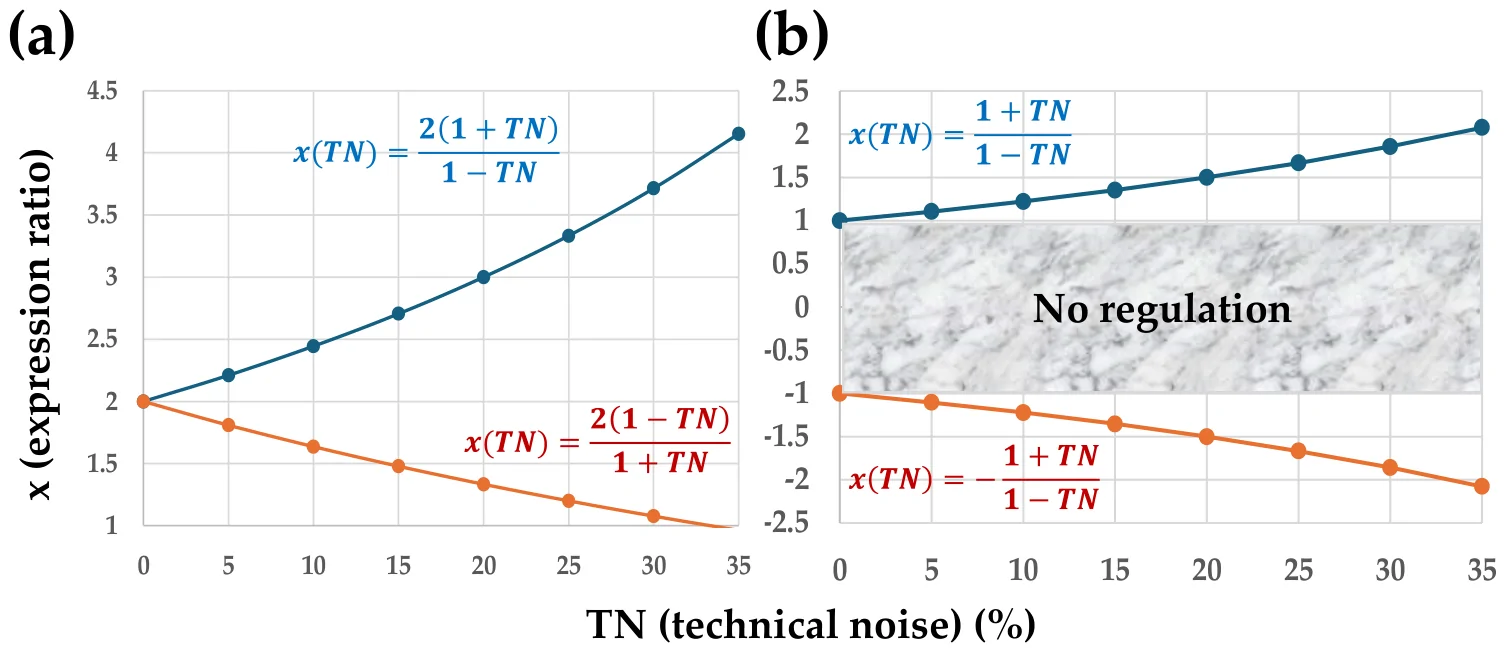
The impact of technical noise on gene expression ratio (negative for downregulation). (**a**) A real two-fold upregulation. (**b**) Real equal expression levels. Note that for 25% noise, the observed expression ratio can be anywhere within the interval [1.33, 3,00], while for 35% noise the interval is [0.96, 4.15]. Because of the technical noise, not all regulated genes are identified, while the regulation of others might be exacerbated. Over 20% noise, expression equality might be turned into a significant up- or downregulation.

Almost all our experimental studies with qRT-PCR, microarrays or NextGen RNA-sequencing started with profiling technical replicas to determine the technical noise of the method. We found that all microarray and sequencing platforms were affected by 25–35% technical noise when respecting the manufacturer protocol, and even qRT-PCR, the so-called “golden standard” for gene expression profiling, is affected by noise [318] (~12% Iacobas, unpublished results). Figure 2 shows how the observed expression ratio of a real 2× upregulation and of a real expression equality are affected by the technical noise, potentially producing false negative or false positive results. Compared to the real expression ratio, x_real_, the observed expression ratio x(TN) is somewhere (nobody knows exactly where) between x_real_(1 − TN)/(1 + TN) and x_real_(1 + TN)/(1 − TN). Now imagine what different results two distinct platforms and/or protocols used to profile the same samples (minimal biological differences) might produce. The differences would be even larger if samples were collected from different animals.

Nevertheless, although a prerequisite of (in)validating regulation of one or a few key genes out of tens of thousands quantified by a high-throughput (microarray or RNA-sequencing) platform, qRT-PCR has no statistical say for the entire transcriptome where up to 5% errors are usually accepted. Therefore, if strictly needed, results of a high-throughput platform should be in principle checked only with another high-throughput platform using technical replicas of the investigated specimens. However, we do not recommend such validation, not only because it doubles the cost and duration of the experiments, but because the conclusion is affected by the combination of the technical noises of the two platforms.

The real value of a high-throughput platform is not with identifying regulation of a few genes, but with the regulation of large groups of genes responsible for a particular biological process. As such, even 5 out of 100 detected regulations might be false hits, and there is still enough information to decide about the transcriptomic alteration of that pathway.

Owing to lower price and the ability to optimize the protocol, we preferred Agilent two-color microarrays [319], even though microarrays profiled redundantly only the 44 k/68 k transcript variants printed on the slide.

Nevertheless, one very important challenge of any optics-based transcriptomic experiment is the choice of fluorescent tags to be efficiently attached to nucleotides used in the reverse transcription. In our experiments, we used both the pair of organic cyanine dyes Cy3 (green emission)/Cy5 (red emission) and the pair Alexa Fluor^®^ 555 (green)/Alexa Fluor^®^ 647 (red). The cyanide pair is affected by different stability and fluorescence intensity of the two fluorophores. While the Alexa pair has the stability advantage across multiple scanning, the cyanides provide a slightly better foreground-to-background ratio [320]. Because of the differences between the green and red dyes, the traditional protocol recommends either profiling each sample twice by swapping the dyes or using a red-labeled universal reference to refer to both tissues to be compared [321]. Each of these recommendations doubles the experimental costs either by profiling the same sample twice or by using a totally uninteresting specimen (the reference). Initially, we prepared a large amount of reference mouse RNA prepared from ten adult mouse tissues (aorta, brain, heart, kidney, liver, lung, ovary/testicles, spleen, and stomach—equal amounts from males and females) to ensure the best coverage of the expressed genes. Later, we avoided such waste by adopting the “multiple yellow strategy” where biological replicas were hybridized against each other on two-color microarray slides. Our strategy eliminates the bias between the two dyes because, instead of red-to-green ratio, we considered red-to-red and green-to-green by normalizing the net signal of any transcript to the median expression of all transcripts in that microarray channel like in a single-color experiment.

### 5.2. Analysis Issues

Traditional analysis is limited to characterizing the transcriptome only by the expression levels of individual genes, like reducing the blueprint of a supercomputer to a list of the electronic components, ignoring how these components are wired and what voltages are applied to each of them. Therefore, we developed the GFP that characterizes each transcript gene by the independent measures “*AVE*” (average expression level), “*REV*” (relative expression variation) and “*COR*” (expression correlation with each other gene across biological replicates) (defined in Appendix A). Compared to traditional analysis limited to only the average expression levels *AVE* of N (>10,000) genes, our strategy also considers N *REV*s and N(N-1)/2 *COR*s (i.e., the entire information provided by the gene expression experiment), thus increasing the amount of data by several orders of magnitude.

The additional independent measures, “*REV*” and “*COR*”, provide valuable biological insights on cellular transcriptomics. “*REV*” (the mid-interval of the chi-square estimate of the coefficient of variation) quantifies through the derived measure “*REC*” (Relative Expression Control) the strength of the homeostatic mechanism to limit the random fluctuation of the gene expression resulting from the stochasticity of the chemical reactions involved in gene transcription. High positive *REC*s indicate genes that are critical for cell physiology, while large negative *REC*s point to genes used as vectors of adaptation to a continuously changing environment [29]. By comparing the *REC* of the disease state with the corresponding control, one determines cell priority changes in controlling expression of important genes.

“*COR*” is the pair-wise Pearson correlation coefficient of the (log_2_) expression levels of two genes across biological replicates. “*COR*” analysis determines the statistically significant transcriptomic networks of functional pathways in the actual condition and their remodeling during the disease development or in response to a treatment. Traditional correlation network analyses [322,323,324] cluster the genes according to their expressions changes in time series, various experimental conditions or other unmatched samples, allegedly to reveal system-level properties of prognostic genes. For instance, Oldham et al. [325] clustered the genes expressed in caudate nucleus, cerebellum, primary motor cortex, and prefrontal cortex in 36 healthy and 44 patients across five grades of Huntington Disease, matched as age and sex. However, we believe that the (dis)similar regulation of two genes is not fixed for all biological systems but dependent on local conditions and therefore subject to remodeling.

*COR*-determined networks are closer to the biological reality than the constructed functional pathways by text-mining software like Pathway Analysis [326], DAVID [327], KEGG [328] or GenMapp/MAPPFinder [329], which are the same regardless of race/strain, sex, hormonal activity, age, diet, climate or other influential factors. Moreover, the gene “wiring” of such inferred pathways is unique (no alternative interlinks) and rigid (does not change during progression of the disease or in response to treatment). Importantly, calcium signaling and several other signaling pathways are considered the same in all tissues. Although we recognize that every functional pathway follows a general script, what genes are currently involved and how they are networked might change to fit the local conditions. It is like building similar houses by using the same architectural blueprint, but the locally available bricks and adhesives might be different in different places.

Expression of a gene is usually considered as significantly regulated when comparing two conditions, D (diseased) vs. C (control), if the *p*-value of the heteroscedastic *t*-test of means’ equality is below a certain value (usually 0.05) and the absolute fold-change (|x|) exceeds a pre-established cut-off (usually 1.5× or 2.0×). Instead of the traditional uniform cut-off, we impose a flexible level “*CUT*”, computed for each transcript to incorporate both the biological variability across biological replicates and the technical noises of the probing spots in the used microarrays (see Appendix A). “*CUT*” condition minimizes the number of false positive hits for very unstably expressed genes probed by noisier spots and the false negative hits for very stably expressed genes and low-noise probing spots.

**Figure 3:**
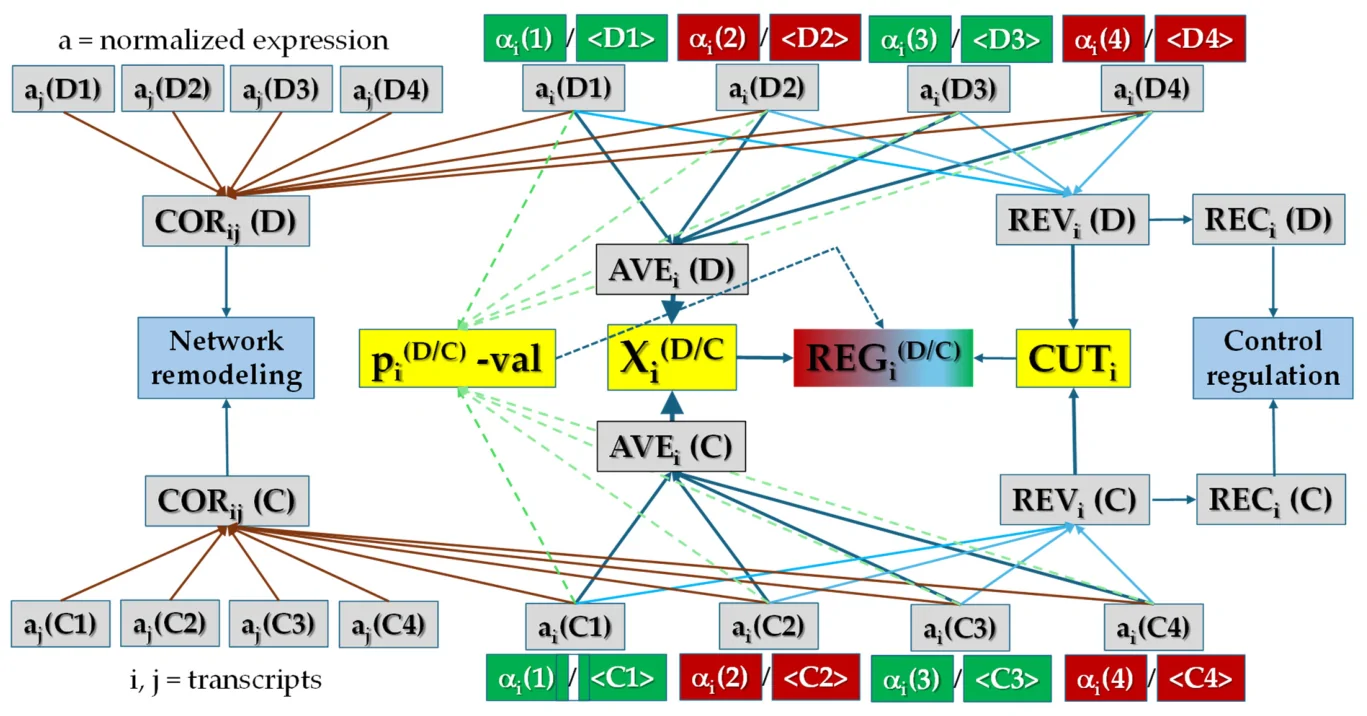
Flow-chart of the mathematical processing of microarray expression data. Background subtracted (net) red/green fluorescence signals “*α*” of the transcript “*i*” (red/green α blocks) are normalized to the median “*α*” in that profiled sample. Normalized expression levels “*a*” are used to compute for every gene *i* in each of the compared conditions “D” (diseased) and “C” (control), the average expression level “*AVE*”, the Relative Expression Variation across biological replicas “*REV*” (and derived measure “*REC*”, Relative Expression Control), and the expression correlation “*COR*” with each other (“*j*”) gene. Results of the two conditions are compared through expression ratio “*x*”, *p*-value “*p-val*” of the heteroscedastic *t-*test of the *AVE* equality and cut-off “*CUT*” of the absolute fold-change |x| to decide about the significance of the expression up-(red) or down(green)-regulation, “*REG*”. The two conditions are also compared in terms of expression control and expression correlation to estimate the changes in the homeostatic mechanisms that limit the random expression fluctuations and the remodeling of the gene networking. Arrows point out from inputs in the analysis to outputs/results.

Figure 3 presents the flow-chart of our computational procedure in microarray experiments.

## 6. Suggestions for the Best Practice of Investigating the Transcriptomic Aspects of Human Neurological Disorders on Animal Models

*Animal choice*: It is preferable to simulate the disease on the closest species to humans. In addition to mice, rats and rabbits, neurological diseases have also been studied in pigs, dogs, cats, horses, goats, and other mammals [330,331,332,333,334,335,336,337,338,339,340,341]. However, non-human primates and large mammals are less affordable and require much more carefully organized housing conditions, as we experienced in studying metabolic syndrome in mongrel dogs [342]. Whenever possible, we suggest using animals from the same litter and of the same sex to minimize the biological variability. It is also important to choose the strain that better recapitulates disease development and response to treatment in humans.

*Animal housing and sacrifice*: Major consideration should be given to housing conditions, limiting sources of stress, and avoiding unnecessary male–male and male–female interactions. Diet, chow and water availability, day–night cycle and light type (no flickering and closer to the natural spectrum) are also very important. Decapitation has lower effects on the nervous system than any other painless sacrifice under anesthesia.

*Sample collection*: Tissue(s) of interest should be dissected as fast as possible and immediately stored in liquid nitrogen or in RNA later [343]. For the female studies we recommend collecting the samples at the same stage of the estrous cycle. Take out 1–2 mm pieces from the apparently most homogeneous regions. Still heterocellular, such tiny pieces are closer to biological reality than monocellular cultures. However, if a single cell phenotype is to be investigated, try to synchronize the cells in the culture and collect the total RNA at distinct stages of the cell cycle. Keep in mind that immortalization has profound effects on cellular processes [344].

*Platform*: Platform choice depends on the study objectives and cost affordability. RNA sequencing can quantify almost all transcripts if you have enough time and money to deepen the reading. However, if the list of quantifiable transcript variants is satisfactory, using Agilent microarrays could be cheaper and less noisy. We recommend profiling four biological replicates of each condition for substantial reduction in the standard error. However, profiling more than four biological replicates is not worth the extra cost and effort because the platform’s technical noise jeopardizes the small increase in statistical relevance. Our optimized wet protocol, “multiple yellow” hybridization strategy, filtering out corrupted spots and close to background fluorescence signals, and normalization to the median net fluorescence in that channel succeeded in reducing the technical noise to 15–20%.

*Analysis*: Regardless of the platform used, we strongly recommend considering all three independent measures to get the full advantage of the information provided by the experiment (four orders of magnitude larger than the traditional analysis). While differential expression analysis is common to all transcriptomic studies, we did not find in the literature alternative approaches to gene expression control and coordination to (in)validate our results. Therefore, we can only speculate about the biological significance of disease-triggered changes in “*REC*” in terms of altered homeostatic control of transcript abundance and “*COR*” as remodeling of gene networks. We recognize the value of the pathway enrichment analysis [345] for biological understanding of the overall disease-triggered alteration of the molecular mechanisms. However, we do believe that WGCNA (weighted gene co-expression network analysis) [346] does not really account for the incredible transcriptomic adaptability of the cell to local constraints, and therefore we recommend its usage only for statistical purposes.

Use of the flexibly determined cut-off “CUT” for the absolute expression ratio minimizes the numbers of both false positive and negative hits. Some authors correct the *p*-value of gene regulation for multiple testing using either Benjamini and Hochberg [347], Benjamini and Yekutieli [348], Bonferroni [349], Hochberg and Benjamini [350], Holm [351], or Hommel [352] methods to allegedly reduce the number of false positives. However, we do not believe that profiling the expressions of distinct genes is multiple testing, since different transcripts hybridize different spotted sequences by forming different sets of H-bonds. Therefore, we apply a Bonferroni-like correction only for groups of spots redundantly probing the same transcript.

*Data deposition, independent validation and integration into metadata*: According to MIAME (minimum information about a microarray experiment) [218], it is required to deposit in publicly accessible databases (like GEO) the used platform, the experimental protocol, the tagged image files (tif) of the scanned microarrays and the raw and normalized expression data. The presented experimental protocol should provide all relevant characteristics of the animals (species, strain, sex, age, treatment, sacrificing protocol etc.), the RNA extraction and tagging procedures and how the expression data were processed. Results of individual experiments can eventually be integrated into metadata and meta-analysis performed. Owing to both technical noise and randomness of the chemical reactions, only the overall regulation of the expression profile, control and correlation of large groups of genes responsible for major functional pathways quantified with a high-throughput platform deserve to be validated as group tendencies through another high-throughput platform.

## 7. Discussion

This EBMC presents issues and sources of errors we faced in decades of profiling the gene expression in cells and tissues collected from animal models of several human neurologic diseases. Online links to publicly available experimental protocols and results of our studies are provided in the References Section for readers interested in reanalyzing our raw data or comparing them with expression profiles of other models (like those mentioned for every disease discussed). Though limited to 12 major pathologies induced in a few strains of mice, rats and rabbits, most of the above discussed aspects are common to transcriptomic studies on animal models of neurological disorders affecting the human nervous system.

Nevertheless, although mimicking the main features of the human disease, an animal model accounts only for a part of the complex, yet not completely known, etiology of that disorder. Even though practically all neuro-diseases are multi-factorial, most of the transgenic animal models had only one gene (termed biomarker) experimentally manipulated (e.g., [353]). Double transgenic (e.g., [354,355]) or even triple transgenic (e.g., [356,357]) animal models are more difficult to produce and therefore less used. Nevertheless, even limited to only one or a few causing factors, an animal model can still help us understand part of the fundamental molecular mechanisms whose alteration causes the disease.

Although the actual technology is far better than what we had in our hands at the time, a good amount of actual knowledge was generated by studies like ours and we believe it is useful to discuss their trustworthiness. Nevertheless, methods such as Frozen Immunolabeled Nuclei Sequencing [358], Single-Cell Combinatorial Fluidic Indexing [359], neuronal cultures in 3D thin gel [360], spatial transcriptomics [361] or Nerve-on-a-Chip [362], to mention just a few, are more suitable for characterizing the high complexity of the nervous structures in a controlled environment. However, even these spectacular new emerging technologies need validation with technical replicates.

## 8. Conclusions

A notable advantage of using animal models is the ability to control most of the regulating factors of the simulated disease in biological replicates and explore various therapeutic avenues. In contrast, beyond several common traits, every human is who s/he is, different from everybody else, even from a monozygotic twin [363,364,365] and introduction of a novel therapy requires a long and complicated process of approvals from the national medical authorities.

## Figures and Tables

**Table 1:** Main features, limitations and biological relevance of our genetically engineered animal models of neurological disorders. GPL indicates the genomic platform (e.g., https://www.ncbi.nlm.nih.gov/geo/query/acc.cgi?acc=GPL10333, accessed on 21 May 2026), while GSE is the number of the expression data series deposited in the NCBI GEO (e.g., https://www.ncbi.nlm.nih.gov/geo/query/acc.cgi?acc=GSE72562, accessed on 21 May 2026). NK = not known, G17 = gestational day 17.

Model	Species	Strain	Sex	Age	Tissue	GPL	Limitations	Relevance	GSE
**Juvenile myoclonic epilepsy (JME)**									
*Brd2^+/−^*	Mouse	C57Bl/6j	Male	Adult	Striata	10,333	heterocellularity	Role of *Brd2*	72,562
			Female						**72** **,** **563**
**Neuropsychiatric lupus erythematosus (NPSLE)**									
*MRL/lpr-Fn14KO*	Mouse	MRL/lpr	Female	Adult	Cortex	10,333	heterocellularity	Pi3k-Akt remodeling	164,140
					Hippocampus			TWEAK effect	169,486
**Oculo-Dento-Digital Displasia (ODDD)**									
*Gja1^−/−^*	Mouse	C57Bl/6j	NK	G17	Whole brain	1698	Regional differences, ODDD not present	Role of *Gja1*	580, 8105, 1954, 8168
					Astrocytes				
*Gja1^+/−^*	Mouse	C57Bl/6j	NK	G17	Whole brain	1698	Regional differences, ODD not present	Role of *Gja1*	580, 8105, 1954, 8168
					Astrocytes				
*siGja1*	Mouse	C57Bl/6j	NK	G17	Astrocytes	5371	ODDD not present	Role of *Gja1*	8168
hGFAP-Cre:Cx43(f/f)	Mouse	C57Bl/6j	NK	neonatal	Whole brain	8928	Regional differences, ODDD not present	Role of *Gja1* Strain and age differences	37,239
		129/SvEv			Astrocytes				
hGFAP-Cre:Cx43(f/f)	Mouse	C57Bl/6j	male	Adult	Whole brain	8928	Regional differences	Role of *Gja1* Strain and age differences	37,239
		129/SvEv			Astrocytes				
**Temporal Lobe epilepsy (TLE)**									
*Gjd2^−/−^*	Mouse	C57Bl/6j	NK	G17	Whole brain	1698	TLE not present	Role of *Gjd2*	6355
*Gjd2^+/−^*									
**X-linked Charcot–Marie–Tooth syndrome type (CMT1X)**									
*Gjb1^−−^*	Mouse	C57Bl/6j	NK	G17	Whole brain	1698	CMT1X not present	Role of *Gjb1*	1954, 6355

**Table 2:** The main features, limitations and biological relevance of our animal models with induced neurological diseases. GPLxxxx indicates the genomic platform (e.g., https://www.ncbi.nlm.nih.gov/geo/query/acc.cgi?acc=GPL5371, accessed on 21 May 2026), while GSE is the number of the expression data series deposited in the NCBI GEO (e.g., https://www.ncbi.nlm.nih.gov/geo/query/acc.cgi?acc=GSE107725, accessed on 21 May 2026). NK = not known, G29 = gestational day 29, ↓ = downregulation, ↑ = increased abundance.

Model	Species	Strain	Sex	Age	Tissue	GPL	Limitations	Relevance	GSE
**Catamenial epilepsy/menstrual seizures (CE)**									
OVX-KA	Rat	Sprague-Davley	Female	Adult	Dentate gyrus (DG)	14,745	Forced menopause, not the cause in women	Protective role of estradiol replacement	107,725, 60,013
**Cerebral malaria (CM)**									
*Plamodium berghei-ANKA*	Mouse	C57Bl/6j	Female	Adult	Whole brain	5371	Regional differences	Putative targets for CM therapy	24,086
**Cerebral palsy (CP)**									
Betamethasone	Rabbit	New Zeeland	NK	G29	Forebrain	7083	Favoring but not the CP cause in humans	Hypomyelination, altered gene networks	44,610
**Glaucoma (GL)**									
ONC	Rat	Lister hooded	Female	Adult	Retina	14,745	Rarely the GL cause in humans	RGS degeneration, altered gene networks	133,563
**Infantile spasms (IS)**									
Two-hit IS (betamethasone + NMDA)	Rat	Sprague-Davley	Female, Male	Adult	Hypothalamus arcuate nuclei, Hypothalamus paraventricular nuclei	14,745	Prenatal betamethasone favoring but not most frequent cause of IS in humans	Effects of betamethasone, sex dichotomy, regional differences, altered synaptic transmission, remodeling of gene networks benefits of ACTH and PMX53 treatments	124,613, 84,585, 124,615, 123,721, 128,090, 128,091
**Intraventricular hemorrhage (IVH)**									
IVH-glycerol	Rabbit	New Zeeland	NK	G29	Microglia, oligodendrocyte precursor cells	25,392	Preterm birth favoring but not the frequent IVH cause in humans	Role of PPAR-γ, altered gene networks	182,718
**Multiple sclerosis (MS)**									
AT-EAE	Mouse	SJL/j	Female	Adult	Spinal cord	1698	Not the real cause of MC in humans	*Gja1* ↓, monocytes ↑, altered gene networks	2446

## Data Availability

No new data were created or analyzed in this study. Data sharing is not applicable to this article.

## Abbreviations

The following abbreviations are used in this manuscript:

AT-EAE	Adoptive transfer experimental autoimmune encephalomyelitis
CE	Catamenial epilepsy
cKO	Conditional knockout
CMT1X	X-linked Charcot–Marie–Tooth syndrome
CM	Cerebral malaria
CP	Cerebral palsy
DG	Dentate gyrus
EBMC	Experience-based methodological commentary
FDA	(US) Food and Drug Administration Agency
GEO	Gene Expression Omnibus
GSE	Gene expression series
GL	Glaucoma
HACN	Hypothalamic arcuate nucleus
HPVN	Hypothalamic paraventricular nucleus
IACUC	Institutional Animal Care and Use Committee
IRB	Institutional Review Board
IS	Infantile spasms
IVH-glycerol	Intraventricular hemorrhage glycerol rabbit model
JME	Juvenile myoclonic epilepsy
MIAME	Minimum information about a microarray experiment
MS	Multiple sclerosis
NCBI	National Center for Biotechnology Information
NMDA	N-methyl-D-aspartic acid
NPSLE	Neuropsychiatric lupus erythematosus
ODDD	Occulo-dento-digital dysplasia
ONC	Oligodendrocyte precursor cells
OPC	Optic nerve crush
OVX-KA	Ovariectomized wih kainic acid induction of epilepsy
qRT-PCR	Quantitative Reverse Transcription Polymerase Chain Reaction
RGC	Retinal ganglion cell
TLE	Temporal lobe epilepsy
TTX	Tetrodotoxin

## Appendix A

### Appendix A.1. The Three Independent Measures of the Gene Expression

∀i,j=1 ÷N  (genes)    &       ∀c=condition/region

(A1) $${AVE}_{i}^{\left(c\right)}\equiv \;\frac{1}{4}\sum_{k=1}^{4}\left({\frac{\propto_{i;k}^{\left(c\right)}}{\langle \alpha_{i;k}^{\left(c\right)}\rangle }}_{i;k}^{\left(c\right)}\right)\;\;with\;a_{i}^{\left(c\right)}=\frac{1}{4}\sum_{k=1}^{4}\propto_{i;k}^{\left(c\right)}\;$$

(A2) $${REV}_{i}^{\left(c\right)}\equiv \;\frac{\sigma_{i}^{\left(c\right)}}{2{AVE}_{i}^{\left(c\right)}}\left(\sqrt{\frac{r_{i}}{\chi^{2}\left(\beta ;r_{i}\right)}}-\sqrt{\frac{r_{i}}{\chi^{2}\left(1-\beta ;r_{i}\right)}}\right)$$

where: σ = standard deviation, *r* = number of freedom degrees, χ2 = chi-square score

(A3) $${COR}_{i,j}^{\left(c\right)}\equiv \;correl\;\left(\log_{2}\left({\frac{\propto_{i;k}^{\left(c\right)}}{\langle \alpha_{i;k}^{\left(c\right)}\rangle }}_{i;k}^{\left(c\right)}\right),\;\log_{2}\left({\frac{\propto_{j;k}^{\left(c\right)}}{\langle \alpha_{j;k}^{\left(c\right)}\rangle }}_{i;k}^{\left(c\right)}\right)\right);\;k=1÷4$$

### Appendix A.2. Significant Expression Regulation

Absolute fold-change and *p*-value composite criterion:

∀i,j=1 ÷N  (genes)    &       C=control, D=disease 

(A4) $$\left\{\begin{array}{c} \left|x_{i}^{\left(D/C\right)}\right|>{CUT}_{i}^{\left(D/C\right)}\\ p_{i}^{\left(D/C\right)}<0.05 \end{array}\right.\;,\;where:$$

(A5) $$x_{i}^{\left(D/C\right)}=\left\{\begin{array}{c} \frac{{AVE}_{i}^{\left(D\right)}}{{AVE}_{i}^{\left(C\right)}}\;\;\;\;\;,\;\;\;\;if\;{AVE}_{i}^{\left(D\right)}>{AVE}_{i}^{\left(C\right)}\;\\ -\frac{{AVE}_{i}^{\left(C\right)}}{{AVE}_{i}^{\left(D\right)}},\;\;\;if\;{AVE}_{i}^{\left(D\right)}\leq {\;AVE}_{i}^{\left(C\right)} \end{array}\right.$$

(A6) $${CUT}_{i}^{\left(D/C\right)}=1+\sqrt{\frac{2}{100}\left({\left({REV}_{i}^{\left(C\right)}\right)}^{2}+{\left({REV}_{i}^{\left(D\right)}\right)}^{2}\right)}$$
